# Thermodynamic Limits of Fault-Tolerant Quantum Computing Beyond the Weak-Coupling, Quasistatic Regime

**DOI:** 10.3390/e28050546

**Published:** 2026-05-11

**Authors:** Mrittunjoy Guha Majumdar

**Affiliations:** 1 Department of Physics, Amrita Vishwa Vidyapeetham, Amritapuri, Vallikavu, Karunagappally, Kollam District, Kerala 690525, India; mrittunjoy@dl.amrita.edu; 2 School of Artificial Intelligence, Amrita Vishwa Vidyapeetham, Mata Amritanandamayi Marg, Faridabad 121002, India; 3 School of Natural Sciences and Engineering, National Institute of Advanced Studies, Bengaluru 560012, India

**Keywords:** thermodynamics, fault tolerance, quantum computing

## Abstract

The standard Landauer bound $W\geq k_{B}T\ln2$ sets the fundamental thermodynamic cost for information erasure under ideal conditions: weak system–bath coupling, quasistatic operation, and equilibrium reservoirs. However, realistic quantum error correction (QEC) operates in a profoundly different regime—finite-time syndrome extraction, strong coupling to cryogenic environments, and non-equilibrium dynamics. Here, we develop a unified thermodynamic framework for fault-tolerant quantum computing that incorporates corrections beyond the ideal Landauer limit. We derive a generalized bound on the heat dissipation per QEC cycle: $Q_{\min}\geq k_{B}T\ln2+k_{B}T\Delta I_{SB}+\frac{\hbar }{\tau }$, and scaling this result to large-scale quantum computers, we find that the total heat load grows polynomially with code distance but remains in the nanowatt range for million-qubit systems—well within the cooling power of modern dilution refrigerators. Applying our model to superconducting qubit architectures, we show that while strong coupling can add up to ∼20% to the ideal cost, finite-time effects contribute approximately 0.55% at 100 ns and 5.5% at 10 ns reset operations. Our results establish that the true thermodynamic cost of fault tolerance, while exceeding the naive Landauer estimate, does not pose a fundamental obstacle to scalability; the dominant engineering challenges lie in the heat load of control electronics and wiring, not in the fundamental dissipation of qubit reset.

## 1. Introduction

Landauer’s principle establishes a fundamental link between information and thermodynamics: erasing one bit of information necessarily dissipates at least $k_{B}T\ln2$ of heat into the environment [1]. This bound was originally derived for a system weakly coupled to an equilibrium heat bath, undergoing a quasistatic (infinitely slow) erasure process that leaves the bath unchanged. Experimental verifications in classical stochastic systems have confirmed the bound under near-reversible conditions [2,3]. The quantum generalization, obtained from unitary evolution of the joint system–bath state, takes the form $\beta \Delta Q=\Delta S_{S}+I\left(\rho_{S}^{\prime }:\rho_{E}^{\prime }\right)+D(\rho_{E}^{\prime }\left|\right|\rho_{E})\geq \Delta S_{S}$ [4]. Here, $\Delta S_{S}$ is the system’s entropy change, *I* is the mutual information quantifying residual correlations between the system and bath after the process, and *D* is the relative entropy measuring how far the bath has been driven from its initial thermal equilibrium. Both *I* and *D* are non-negative, so the inequality saturates—i.e., the Landauer bound becomes an equality—only under three simultaneous conditions: (i) no final system–bath correlations (I=0), (ii) the bath remains exactly thermal (D=0), and (iii) the dynamics is reversible (quasistatic). These conditions define an idealized regime that is systematically violated in any practical implementation of quantum information processing. Fault-tolerant quantum computing overcomes physical errors by encoding logical information redundantly across many physical qubits [5,6], a principle that extends to architectures for quantum network [7].

In leading architectures such as the surface code, error correction proceeds in repeated cycles, each consisting of three steps: entangling ancilla qubits with data qubits to extract error syndromes, measuring the ancillas to obtain classical syndrome information, and resetting the ancillas to their ground state for the next cycle [8,9]. The reset step is an instance of information erasure: each cycle resets approximately $N_{\mathrm{phys}}/2$ ancillas, each carrying up to one bit of syndrome information. Under the ideal (weak-coupling, quasistatic) conditions, the heat generated per cycle is, therefore, at least $\left(N_{\mathrm{phys}}/2\right)k_{B}T\ln2$. This ideal cost sets a baseline, but it is achieved only in the limit of infinitely slow, reversible operations with no residual correlations—conditions that are incompatible with the fast, noisy, and strongly coupled environment of a real quantum processor.

Realistic quantum computers operate in a profoundly different regime. Finite-time operation is essential because error suppression demands rapid cycle times—current superconducting qubit systems target 100 ns, with roadmaps aiming for 10 ns [10]. Strong coupling is deliberately engineered: qubits are coupled to control lines, readout resonators, and the cryogenic bath with interaction strengths g∼10–100 MHz; at a typical operating temperature of 20 mK, the thermal energy scale $k_{B}T/h\approx 400$ MHz, so the coupling can be comparable to or even exceed thermal energy [11]. Moreover, continuous syndrome measurement and feedback drive the environment into a non-equilibrium steady state, creating local heating that can feed back into error rates. A dynamical phase transition driven by Landauer heating has been identified: when the cooling power of the refrigerator falls below a threshold, the system enters an “unbounded-error phase” where temperature runs away and error rates exceed the fault-tolerance threshold [12]. That analysis, however, assumed the ideal Landauer cost per erasure and treated the system–bath coupling as weak—precisely the assumptions that are violated in practice. Concurrently, a finite-time Landauer principle beyond weak coupling has been derived, showing that optimal erasure protocols in the slow-driving regime incur additional dissipation characterized by a thermodynamic length, and it identifies the Planckian time $\tau_{P}∼\hbar /k_{B}T$ as the fundamental shortest timescale for information erasure [13]. Thus, a comprehensive assessment of the thermodynamic cost of fault-tolerant quantum computing must account for both finite-time and strong-coupling corrections beyond the ideal Landauer bound—a task that the present paper undertakes.

This paper advances the following thesis: The true thermodynamic cost of fault-tolerant quantum computing exceeds the standard Landauer bound by quantitatively significant corrections arising from finite-time operation and strong system–bath coupling. We derive a unified bound incorporating these corrections, scale it to large-scale architectures, and evaluate its implications for superconducting qubit systems. Section 2 reviews the necessary background on Landauer’s principle and the threshold theorem. Section 3 develops the finite-time correction using geometric thermodynamics. Section 4 derives the strong-coupling correction via the Hamiltonian of mean force. Section 5 unifies these corrections and scales them to fault-tolerant architectures. Section 6 applies the model to superconducting qubit systems with quantitative estimates. Section 5 discusses mitigation strategies and open questions.

## 2. Background

### 2.1. Quantum Landauer Principle

Let the system *S* be described by a finite-dimensional Hilbert space $H_{S}$ and the environment E by $H_{E}$. The joint Hamiltonian is $H_{\mathrm{tot}}=H_{S}+H_{E}+H_{SB}$, where $H_{SB}$ is the interaction. The environment is initially in a Gibbs state at inverse temperature $\beta =1/(k_{B}T)$:

$$\rho_{E}=\frac{e^{-\beta H_{E}}}{Z_{E}},\;Z_{E}={\mathrm{Tr}}_{E}e^{-\beta H_{E}}.$$

The initial system state is arbitrary $\rho_{S}$, and the total initial state is assumed to be a product:

$$\rho_{SE}\left(0\right)=\rho_{S}⊗\rho_{E}.$$

The joint system evolves unitarily for time *t*:

$$\rho_{SE}\left(t\right)=U\left(t\right)\left(\rho_{S}⊗\rho_{E}\right)U{\left(t\right)}^{†},\;U\left(t\right)=e^{-iH_{\mathrm{tot}}t/\hbar }.$$

Define the entropy change of the system:

$$\Delta S_{S}=S\left(\rho_{S}\right)-S\left(\rho_{S}\left(t\right)\right),\;S\left(\rho \right)=-\mathrm{Tr}\left(\rho \ln\rho \right),$$

and the heat transferred to the environment as the change in its internal energy (since no work is done on the bath):

$$\Delta Q=\mathrm{Tr}\left[H_{E}\left(\rho_{E}\left(t\right)-\rho_{E}\right)\right].$$

The quantum Landauer bound is a direct consequence of the unitary evolution and the non-negativity of the quantum relative entropy. One obtains [4]:

(1) $$\beta \Delta Q=\Delta S_{S}+I\left(\rho_{S}\left(t\right):\rho_{E}\left(t\right)\right)+D\left(\rho_{E}\left(t\right)∥\rho_{E}\right)\geq \Delta S_{S},$$

where

- $I\left(\rho_{S}:\rho_{E}\right)=S\left(\rho_{S}\right)+S\left(\rho_{E}\right)-S\left(\rho_{SE}\right)$ is the mutual information, quantifying total correlations (classical and quantum) between the system and the environment.
- $D\left(\rho_{E}\left(t\right)∥\rho_{E}\right)=\mathrm{Tr}\left[\rho_{E}\left(t\right)\left(\ln\rho_{E}\left(t\right)-\ln\rho_{E}\right)\right]$ is the relative entropy, measuring how far the environment has been driven from equilibrium.

Both terms are non-negative, and they vanish simultaneously if, and only if:

1. The final state is a product: $\rho_{SE}\left(t\right)=\rho_{S}\left(t\right)⊗\rho_{E}\left(t\right)$ (no correlations),
2. The environment returns exactly to its initial thermal state: $\rho_{E}\left(t\right)=\rho_{E}$.

These conditions are met only in the limit of a quasistatic (infinitely slow) process with weak coupling ($H_{SB}\to 0$). For the erasure of one bit, where the system is reset from a maximally mixed state $\rho_{S}=\frac{1}{2}1_{2}$ to a pure ground state $\rho_{S}\left(t\right)=\left|0\rangle \langle 0\right|$, the entropy change is $\Delta S_{S}=\ln2$. Thus, the minimal heat satisfies $\Delta Q\geq k_{B}T\ln2$, with equality only in the idealized reversible, weak-coupling, quasistatic limit.

### 2.2. Fault-Tolerant Quantum Computing and the Threshold Theorem

Fault-tolerant quantum computing (FTQC) provides a method to perform arbitrarily long quantum computations despite the presence of noise, provided the noise level is below a certain threshold. The fundamental result is the threshold theorem, which we state in a simplified form:

**Theorem** **1** (Threshold Theorem [5,6])**.** *There exists a constant $p_{\mathrm{th}}>0$ such that, if the physical error probability per gate or per time step is less than $p_{\mathrm{th}}$, then any quantum circuit of length L can be simulated with a probability of error at most ϵ using a number of physical qubits and gates that scales polynomially in L and log(1/ϵ)*.

For the surface code—a leading topological quantum error-correcting code—the threshold has been numerically estimated to be $p_{\mathrm{th}}\approx 1\%$ [9]. The thermodynamic framework presented here can, in principle, be extended to other topological codes, such as non-CSS color codes on 2D lattices [14], which may exhibit different resource costs. The surface code is defined on a two-dimensional lattice of physical qubits. A logical qubit is encoded using a distance-*d* surface code, which employs $N_{\mathrm{phys}}=2d^{2}-1$ physical qubits (for the rotated code). The code distance *d* determines the number of correctable errors: any error affecting fewer than (d−1)/2 physical qubits can be corrected. Error correction proceeds in discrete cycles. Each cycle consists of:

1. Syndrome extraction: For each stabilizer generator (a Pauli operator that commutes with the code’s logical operations), an ancilla qubit is entangled with a set of data qubits via a sequence of controlled-NOT gates. The state of the ancilla then encodes the eigenvalue (±1) of the stabilizer.
2. Measurement: Each ancilla is measured in the computational basis, yielding a classical syndrome bit.
3. Reset: Each ancilla is reset to its ground state |0〉 in preparation for the next cycle.

The reset step is an information-erasure process: each ancilla initially contains up to one bit of syndrome information (the measurement outcome). Under ideal conditions (weak-coupling, quasistatic operation), the heat generated per reset is at least $k_{B}T\ln2$, so the total heat per cycle is at least $\left(N_{\mathrm{phys}}/2\right)k_{B}T\ln2$. The cycle time $\tau_{\mathrm{cycle}}$ must be chosen such that the probability of an error occurring during the cycle is below the threshold. Let $\tau_{\mathrm{gate}}$ be the duration of a single elementary gate (e.g., a CNOT). The syndrome extraction circuit contains O(d) gates, so $\tau_{\mathrm{cycle}}∼O\left(d\right)\tau_{\mathrm{gate}}$. A more precise condition is derived from the requirement that the logical error probability per cycle, which scales as ${\left(p_{\mathrm{err}}/p_{\mathrm{th}}\right)}^{(d+1)/2}$, remains sufficiently small. This leads to a scaling relation:

(2) $$\tau_{\mathrm{cycle}}≲\frac{p_{\mathrm{th}}}{p_{\mathrm{err}}}\tau_{\mathrm{gate}},$$

which reflects the trade-off: faster gates (small $\tau_{\mathrm{gate}}$) allow a higher physical error rate $p_{\mathrm{err}}$ while keeping the logical error rate fixed. Various platform-specific gate implementations, such as those based on the exchange interaction [15,16], may exhibit different noise and dissipation characteristics and represent a promising direction for future investigation.

### 2.3. Landauer Heating in Fault-Tolerant Systems: Dynamical Model and Phase Transition

The heat dissipated during reset elevates the local temperature of the quantum processor. Since the error probability $p_{\mathrm{err}}$ is a monotonically increasing function of temperature (due to thermal excitation of qubits and increased noise in control electronics), a positive feedback loop emerges: higher temperature increases errors, which, in turn, may require more frequent resetting or longer cycles, generating even more heat. This feedback can be described by a coupled system of differential equations for the temperature T(t) and the error rate. Following [12], we consider a lumped-element thermal model. Let *C* be the total heat capacity of the qubit chip (assumed constant over the small temperature range of interest). The heat generation rate from ancilla reset is:

$${\dot{Q}}_{\mathrm{gen}}=\frac{N_{\mathrm{anc}}}{\tau_{\mathrm{cycle}}}\cdot k_{B}T\ln2,$$

where $N_{\mathrm{anc}}\approx N_{\mathrm{phys}}/2$ is the number of ancillas reset per cycle. This expression assumes the ideal Landauer cost; corrections will be added later. The cooling power provided by the dilution refrigerator is typically proportional to the temperature difference:

$${\dot{Q}}_{\mathrm{cool}}=\frac{T-T_{\mathrm{bath}}}{R_{\mathrm{th}}},$$

where $R_{\mathrm{th}}$ is the effective thermal resistance between the qubit chip and the refrigerator’s mixing chamber (held at base temperature $T_{\mathrm{bath}}$). The cooling time constant is $\tau_{\mathrm{cool}}=CR_{\mathrm{th}}$. The net heating rate is ${\dot{Q}}_{\mathrm{gen}}-{\dot{Q}}_{\mathrm{cool}}$, and since ${\dot{Q}}_{\mathrm{net}}=C\dot{T}$, we obtain:

(3) $$\dot{T}=\frac{N_{\mathrm{anc}}k_{B}T\ln2}{\tau_{\mathrm{cycle}}C}-\frac{T-T_{\mathrm{bath}}}{\tau_{\mathrm{cool}}}.$$

This is a first-order linear differential equation for T(t) (with the coefficient of *T* on the right-hand side being constant). Its solution is:

(4) $$\begin{array}{c} T\left(t\right)=T_{\mathrm{bath}}\frac{1/\tau_{\mathrm{cool}}}{1/\tau_{\mathrm{cool}}-N_{\mathrm{anc}}k_{B}\ln2/\left(\tau_{\mathrm{cycle}}C\right)}\\ +\left(T\left(0\right)-T_{\mathrm{bath}}\frac{1/\tau_{\mathrm{cool}}}{1/\tau_{\mathrm{cool}}-N_{\mathrm{anc}}k_{B}\ln2/\left(\tau_{\mathrm{cycle}}C\right)}\right)e^{\left(\frac{N_{\mathrm{anc}}k_{B}\ln2}{\tau_{\mathrm{cycle}}C}-\frac{1}{\tau_{\mathrm{cool}}}\right)t}. \end{array}$$

The temperature diverges exponentially if the exponent is positive, i.e., if:

(5) $$\frac{N_{\mathrm{anc}}k_{B}\ln2}{\tau_{\mathrm{cycle}}C}>\frac{1}{\tau_{\mathrm{cool}}}.$$

This inequality defines the boundary of a dynamical phase transition. When it holds, the system enters an “unbounded-error phase”: the temperature rises without bound, error rates become arbitrarily large, and fault tolerance is lost. Conversely, when the cooling rate dominates, the system reaches a stable steady-state temperature:

$$T_{\mathrm{steady}}=\frac{T_{\mathrm{bath}}}{1-\frac{N_{\mathrm{anc}}k_{B}\ln2}{\tau_{\mathrm{cycle}}C}\tau_{\mathrm{cool}}}.$$

The analysis leading to Equation (5) makes two crucial idealizations: (i) it assumes the weak-coupling regime, where the Landauer cost is exactly $k_{B}T\ln2$ and the cooling power is given by the simple linear law ${\dot{Q}}_{\mathrm{cool}}=\left(T-T_{\mathrm{bath}}\right)/R_{\mathrm{th}}$; (ii) it assumes that the reset operation is performed quasistatically, so that no additional finite-time dissipation occurs. In realistic superconducting qubit systems, both assumptions are violated: the system–bath coupling is strong (g∼10–100 MHz, comparable to $k_{B}T/h$), and reset must be fast (10–100 ns) to keep up with the error rate. The present work generalizes the thermal model by incorporating finite-time and strong-coupling corrections to the Landauer bound, thereby providing a more accurate determination of the true thermodynamic limits of fault-tolerant quantum computing.

### 2.4. Finite-Time Corrections to the Landauer Bound

The minimum time required for any quantum state transformation is bounded by the Mandelstam–Tamm relation [17]. For a qubit reset from a maximally mixed state to the ground state, the energy uncertainty ΔE is at most the qubit energy splitting $E=\hbar \omega_{q}$. This gives a lower bound on the reset time τ:

$$\tau \geq \frac{\hbar \arccos(|\langle \psi (0)|\psi (\tau )\rangle |)}{\Delta E}∼\frac{\hbar }{E}.$$

For typical superconducting qubits ($\omega_{q}/2\pi =5$ GHz), this fundamental limit is ∼30 ps, far below the cycle times of interest (10–100 ns). Hence, the process is not speed-limited in the sense of time–energy uncertainty. A general result from the quantum speed limit and finite-time thermodynamics [17,18] states that any process completed in a finite time τ incurs an excess dissipation above the reversible limit that scales as

$$Q_{\mathrm{FT}}≳\frac{\hbar }{\tau }.$$

This bound follows from the quantum speed limit and has dimensions of energy (since ℏ/τ has dimensions of action/time = energy). It is the minimal possible excess heat for a reset operation; any realistic protocol will dissipate at least this amount.

### 2.5. Strong-Coupling Corrections

The standard Landauer derivation assumes the system–bath interaction Hamiltonian $H_{SB}$ is negligible in the initial and final states. In superconducting circuits, however, qubits are deliberately coupled to control lines and readout resonators with interaction strengths g∼10–100 MHz [11]. At T=20mK, $k_{B}T/h\approx 400\;\mathrm{MHz}$, so *g* can be comparable to or exceed thermal energy. When $H_{SB}$ is non-negligible, the free energy change of the system is no longer simply $\Delta F_{S}$. The correct thermodynamic potential is the *Hamiltonian of mean force* [19,20]:

(6) $$H_{S}^{*}\left(\beta \right)=-\frac{1}{\beta }\ln\left(\frac{{\mathrm{Tr}}_{E}e^{-\beta H_{\mathrm{total}}}}{Z_{E}}\right).$$

The effective free energy of the system becomes $F_{S}^{*}=-\beta^{-1}\ln{\mathrm{Tr}}_{S}e^{-\beta H_{S}^{*}}$, and the entropy change splits into system and correlation contributions:

(7) $$\Delta S=\Delta S_{S}+\Delta S_{\mathrm{corr}}.$$

The strong-coupling generalization of Landauer’s principle is given as [21]:

(8) $$\Delta Q\geq -\Delta F_{S}^{*}=\Delta U_{S}^{*}-T\Delta S_{S}^{*}.$$

The correlation entropy $S_{\mathrm{corr}}=S(\rho_{S}\left|\right|\rho_{S}^{\mathrm{th}})$, where $\rho_{S}^{\mathrm{th}}$ is the reduced state of the system if it were thermalized with the bath under full coupling, accounts for system–bath entanglement and information. For erasure of a qubit, the additional heat cost due to strong coupling is:

(9) $$Q_{\mathrm{SC}}=k_{B}T\;\Delta I_{SB},$$

where $\Delta I_{SB}$ is the change in mutual information between the system and bath during the process (in nats). This term quantifies the extra dissipation required to dismantle system–bath correlations. For a transmon qubit coupled to a readout resonator with coupling *g* and resonator damping rate κ, the steady-state mutual information can be estimated. In the strong-dispersive regime (χ≫κ), measurement extracts significant information, leaving residual correlations [22]. The interaction Hamiltonian in the dispersive regime is:

(10) $$H_{\mathrm{int}}=\hbar \chi \;a^{†}a\;\sigma_{z},$$

where $\chi =g^{2}/\Delta$ is the dispersive shift and Δ is the detuning. Measurement projects the qubit state but leaves residual qubit–resonator entanglement due to finite κ. Numerical simulations for typical parameters (g/2π=50MHz,κ/2π=1MHz,χ/2π=5MHz) yield $I_{SB}∼$ 0.1–0.3 bits after measurement [23]. The change in mutual information during reset, $\Delta I_{SB}$, depends on the initial state before reset. For ancillas that were just measured, the initial mutual information is approximately the measurement-induced value. After reset to the ground state with no residual correlations, $I_{SB}^{\mathrm{final}}\approx 0$. Thus:

(11) $$\Delta I_{SB}\approx I_{SB}^{\mathrm{initial}}∼0.2\;\mathrm{bits}=0.2\ln2\approx 0.139\;\mathrm{nats}.$$

This gives:

$$Q_{\mathrm{SC}}=k_{B}T\times 0.139=\left(1.38\times 10^{-23}\;J/K\right)\left(0.02\;K\right)\left(0.139\right)=3.84\times 10^{-26}\;J\approx 0.24\;\mu \mathrm{eV}.$$

Compared to the ideal Landauer cost of 1.19 μeV, this represents a 20% addition. (We emphasize that this value is obtained from the steady-state mutual information after a measurement, which may differ from the dynamical mutual information that builds up and is destroyed during the reset process itself. A full treatment would require solving the time-dependent master equation for the coupled qubit-resonator system during reset, a task beyond the scope of the present work. Hence, the 20% correction should be regarded as an order-of-magnitude estimate; more precise calculations could either increase or decrease this value depending on the protocol and the coupling regime.) We further note that a rigorous non-perturbative treatment (e.g., geometric thermodynamics [13]) would combine finite-time and strong-coupling effects into a single correction, not as a simple sum. Hence, our estimate of $\Delta I_{SB}$ is an approximation, and the additive form in Equation (16) (later replaced by an approximate sum) is not a rigorous bound. The dynamical model of Bilokur et al. [12] assumes weak coupling to the refrigerator. Strong coupling modifies the heat flow equation in two ways:

1. *Increased effective thermal resistance*: Correlations impede heat transfer, effectively reducing the cooling power. The thermal conductance between the qubit and the bath becomes [24]:
  (12) $$G_{\mathrm{eff}}=G_{0}\left(1-\frac{\chi^{2}}{\kappa^{2}+\chi^{2}}\right),$$
  where $G_{0}$ is the weak-coupling conductance. This expression, derived from linear response theory, captures the reduction in heat flow due to correlations, but its validity for the strongly driven, non-equilibrium conditions of rapid QEC cycles is uncertain. A more accurate modeling would require a full counting statistics approach.
2. *Modified temperature dependence*: The qubit’s effective temperature under strong coupling differs from the bath temperature. The steady-state qubit excitation probability is [25]:
  (13) $$p_{e}=\frac{1}{2}\left(1-\frac{\kappa }{\sqrt{\kappa^{2}+4\chi^{2}\langle n\rangle }}\right),$$
  where 〈n〉 is the resonator photon number.

Both effects shift the phase boundary between bounded- and unbounded-error regimes, potentially making the unbounded phase more accessible. For typical parameters, the correction to the critical cooling power is ∼10–20%.

### 2.6. Non-Markovian Effects and Information Backflow

The analysis above assumes a Markovian environment (memoryless). However, cryogenic environments in superconducting circuits can exhibit non-Markovian behavior, especially in “giant atom” architectures where a qubit couples to multiple points on a waveguide [26]. Non-Markovianity enables information backflow: entropy previously dissipated into the bath can temporarily return to the system, leading to transient negative entropy production rates [27]. This backflow modifies the effective Landauer bound for finite-time processes because the system-bath mutual information $I_{SB}$ becomes time-dependent and can oscillate. A rigorous bound incorporating non-Markovianity is given in Ref. [28]:

(14) $$\beta \Delta Q\geq \Delta S_{S}+I\left(\rho_{S}:\rho_{E}\right)+\Phi ,$$

where Φ≥0 is a non-Markovianity quantifier (e.g., the BLP measure). For typical superconducting qubit parameters, the correction Φ is estimated to be of order 0.01–0.05$k_{B}T$, i.e., a few percent of the ideal Landauer cost. Thus non-Markovian effects do not change our qualitative conclusions but may become relevant in ultrastrong coupling or highly structured bath regimes.

## 3. Unified Thermodynamic Bound for Fault-Tolerant Quantum Computing

Combining the ideal Landauer cost, the finite-time penalty, and the strong-coupling correction, we obtain an approximate expression for the heat dissipated per ancilla reset:

$$Q_{\mathrm{reset}}\approx k_{B}T\ln2+\frac{\hbar }{\tau_{\mathrm{reset}}}+k_{B}T\Delta I_{SB}$$

Here, the second term is the minimal excess heat from finite-time operation (quantum speed limit), and the third term is an estimate of the additional cost due to residual system–bath correlations (strong coupling). Because the finite-time term is ≲5.5% of the Landauer cost for $\tau_{\mathrm{reset}}\geq 10$ ns, and the strong-coupling term is ∼20%, the dominant beyond-ideal correction for current architectures is the strong-coupling contribution. We caution that this expression is not a rigorous bound; a full non-perturbative treatment would not separate the two corrections additively. Nonetheless, it provides a useful estimate for the expected dissipation.

In a surface code with distance *d*, the number of physical qubits $N_{\mathrm{phys}}=2d^{2}-1$ (for rotated code) [9]. Each QEC cycle of duration $\tau_{\mathrm{cycle}}$ involves resetting all ancillas (approximately $N_{\mathrm{phys}}/2$ for typical implementations). The total heat generation rate is:

(15) $${\dot{Q}}_{\mathrm{total}}=\frac{N_{\mathrm{phys}}}{2}\cdot \frac{Q_{\mathrm{reset}}}{\tau_{\mathrm{cycle}}}.$$

Substituting our generalized bound and noting $\tau_{\mathrm{reset}}\leq \tau_{\mathrm{cycle}}$:

(16) $${\dot{Q}}_{\mathrm{total}}\approx \frac{N_{\mathrm{phys}}}{2\tau_{\mathrm{cycle}}}\left(k_{B}T\ln2+\frac{\hbar }{\tau_{\mathrm{cycle}}}+k_{B}T\Delta I_{SB}\right).$$

The three terms scale differently with temperature and cycle time:

- Ideal term: $\propto T/\tau_{\mathrm{cycle}}$,
- Finite-time term: $\propto 1/\tau_{\mathrm{cycle}}^{2}$ (independent of *T*),
- Strong-coupling term: $\propto T/\tau_{\mathrm{cycle}}$.

For fault tolerance to be thermodynamically sustainable, the cooling power $P_{\mathrm{cool}}$ of the refrigerator must exceed ${\dot{Q}}_{\mathrm{total}}$. This yields a scalability condition:

(17) $$P_{\mathrm{cool}}\geq \frac{N_{\mathrm{phys}}}{2\tau_{\mathrm{cycle}}}\left(k_{B}T\ln2+\frac{\hbar }{\tau_{\mathrm{cycle}}}+k_{B}T\Delta I_{SB}\right).$$

The right-hand side scales as $N_{\mathrm{phys}}/\tau_{\mathrm{cycle}}$ times the per-reset cost. Since $N_{\mathrm{phys}}\propto d^{2}$ and logical error rate suppression requires $\tau_{\mathrm{cycle}}$ to decrease logarithmically with *d* [29], the heat load grows slightly faster than linearly with code distance.

## 4. Application to Superconducting Qubit Architectures

We specialize to a superconducting transmon qubit system operating at the base temperature of a dilution refrigerator, T=20mK. The qubit energy splitting is taken as E=ℏ×5GHz≈3.3μeV, and the energy relaxation time is $T_{1}=100\;\mu s$, which is typical for state-of-the-art transmons. The cycle time for the surface code is chosen in the range $\tau_{\mathrm{cycle}}=100\;\mathrm{ns}$ (representing current experiments) down to 10ns (a target for future fast gates). Code distances vary from d=3 for a small logical qubit to d=27 for large-scale error suppression, where the physical error probability is suppressed to $p_{\mathrm{err}}∼10^{-12}$. The number of logical qubits in a full-scale computer is taken as $N_{L}=10^{6}$. The strong-coupling correction requires an estimate of the change in system–bath mutual information; we adopt $\Delta I_{SB}=0.2\;\mathrm{bits}=0.139\;\mathrm{nats}$ as a static estimate derived from measurement-induced correlations in the dispersive regime. The ideal Landauer cost for erasing one bit is $Q_{\mathrm{ideal}}=k_{B}T\ln2=1.91\times 10^{-25}\;J=1.19\;\mu \mathrm{eV}$. The finite-time correction, derived from the quantum speed limit, is $Q_{\mathrm{FT}}\left(\tau \right)=\hbar /\tau$. For a reset time τ=100ns, this amounts to $1.05\times 10^{-27}\;J=6.6\times 10^{-3}\;\mu \mathrm{eV}$, which represents only 0.55% of the ideal Landauer cost; even for the aggressive target τ=10ns, the correction grows to 5.5% of the ideal cost, still a small fraction. Consequently, the finite-time term is completely negligible in the overall heat budget. The strong-coupling correction, estimated from the residual mutual information after measurement, gives $Q_{\mathrm{SC}}=k_{B}T\Delta I_{SB}=3.84\times 10^{-26}\;J=0.24\;\mu \mathrm{eV}$, which is about 20% of the ideal cost. Adding the two dominant contributions, the total heat dissipated per ancilla reset is approximately $Q_{\mathrm{reset}}\approx Q_{\mathrm{ideal}}+Q_{\mathrm{SC}}=1.43\;\mu \mathrm{eV}$, i.e., 20% above the ideal Landauer limit. The finite-time correction is too small to affect this sum at the precision considered.

In a surface code of distance *d*, the number of physical qubits is $N_{\mathrm{phys}}=2d^{2}-1$ (rotated code). For the largest distance considered, d=27, we have $N_{\mathrm{phys}}\approx 1458$ and the number of ancillas reset per cycle is $N_{\mathrm{anc}}\approx N_{\mathrm{phys}}/2=729$. The total heat generation rate is ${\dot{Q}}_{\mathrm{total}}=\left(N_{\mathrm{anc}}/\tau_{\mathrm{cycle}}\right)\;Q_{\mathrm{reset}}$. Using the per-reset heat from above, for $\tau_{\mathrm{cycle}}=100\;\mathrm{ns}$, we obtain ${\dot{Q}}_{\mathrm{total}}=729/10^{-7}\;s\times 1.43\times 10^{-25}\;J=1.04\times 10^{-15}\;W=1.04\;\mathrm{fW}$. For the faster cycle time $\tau_{\mathrm{cycle}}=10\;\mathrm{ns}$, the heat load increases by a factor of ten to 10.4fW. Scaling these numbers to a full-scale quantum computer with $N_{L}=10^{6}$ logical qubits (each encoded with distance d=27, giving a total of about $1.5\times 10^{9}$ physical qubits) yields a total heat load of ${\dot{Q}}_{\mathrm{total}}\approx 1.04\;\mathrm{nW}$ at 100ns cycle time and 10.4nW at 10ns cycle time. Because the finite-time correction is negligible, these values are unchanged from estimates that only include the ideal Landauer and strong-coupling terms. Modern dilution refrigerators operating at 20mK provide a cooling power $P_{\mathrm{cool}}∼10\;\mu W$ [30]. Our calculated heat loads for a million-logical-qubit system are in the nanowatt range, i.e., four orders of magnitude below the available cooling power. Even when additional heat sources such as control wiring, readout electronics, and classical control hardware are taken into account, the fundamental thermodynamic cost of ancilla reset—dominated by the ideal Landauer and strong-coupling contributions—remains well within the cooling capacity. Therefore, from the perspective of reset thermodynamics alone, scalability is not constrained by the refrigerator’s power limit.

Although the global heat load is minuscule, local heating in the vicinity of the qubits could, in principle, create temperature gradients that affect coherence and error rates. To assess this, we employ the heat diffusion model of Bilokur et al. [12], which gives the local temperature rise as $\Delta T_{\mathrm{local}}={\dot{Q}}_{\mathrm{local}}/\left(4\pi \kappa_{\mathrm{th}}r\right)$, where $\kappa_{\mathrm{th}}$ is the thermal conductivity of the substrate and *r* is the distance to the heat sink. For a silicon substrate at 20mK, the thermal conductivity is $\kappa_{\mathrm{th}}∼0.1\;W\;m^{-1}\;K^{-1}$ [31]. Taking a single qubit dissipating ${\dot{Q}}_{\mathrm{local}}∼10^{-15}\;W$ and a distance r=1mm to the heat sink, we obtain $\Delta T_{\mathrm{local}}\approx 10^{-15}/\left(4\pi \times 0.1\times 10^{-3}\right)\approx 8\times 10^{-13}\;K$, which is utterly negligible. Even for a hot spot containing $10^{6}$ qubits within a volume of $1\;{\mathrm{cm}}^{3}$, the total dissipated power is ∼10 nW, yielding a local temperature rise $\Delta T_{\mathrm{local}}∼10^{-8}\;K$. Such minuscule gradients pose no threat to qubit coherence or to the fault-tolerance threshold. Consequently, neither global nor local thermodynamic constraints limit the scalability of superconducting quantum computers based on the surface code.

While the global heat load is small, the transfer of heat from the silicon chip to the refrigerator’s mixing chamber is limited by the Kapitza thermal boundary resistance $R_{K}$. At millikelvin temperatures, $R_{K}$ scales as $T^{-3}$ and can dominate over bulk thermal resistance [31]. The heat flow across an interface of area *A* is $\dot{Q}=A\;\kappa_{K}\left(T_{\mathrm{chip}}^{4}-T_{\mathrm{bath}}^{4}\right)$, where $\kappa_{K}$ is the Kapitza conductance. However, precise values of $\kappa_{K}$ for metal-semiconductor interfaces at millikelvin temperatures are not well-characterized and are expected to be orders of magnitude lower than at room temperature, often dominated by the $T^{3}$ scaling of phonon transport. This reveals that the true thermodynamic bottleneck is not solely the refrigerator’s cooling power but also the efficiency of interfacial heat transport. Mitigation strategies include using silver sinters with high specific surface areas (e.g., up to 1.4 m^2^/g) [32] and direct bonding of the chip to the mixing chamber [33].

## 5. Discussion

Our quantitative analysis yields a clear conclusion: the finite-time correction to the Landauer bound for realistic superconducting qubit parameters is seen to be contributing approximately 0.55% at 100 ns and 5.5% at 10 ns reset operations. The strong-coupling correction, estimated from the residual mutual information after measurement, adds approximately 20% to the ideal cost and is the dominant beyond-ideal term. However, even with this correction, the absolute heat load for a million-logical-qubit system remains in the nanowatt range—far below the cooling power of existing cryogenic systems. This suggests that the primary thermodynamic constraint on fault-tolerant quantum computing is not the fundamental dissipation of qubit reset, but rather engineering challenges: heat load from control electronics, wiring thermal conductance, and the need to maintain millikelvin temperatures despite significant power dissipation from classical control systems operating at room temperature. The finite-time corrections, while conceptually interesting, do not pose a practical obstacle. While the finite-time correction is negligible across all practically relevant timescales, the strong-coupling term can become more significant in two regimes:

- *Higher-temperature operation:* At T=100mK, the ideal Landauer cost increases 5×, and the strong-coupling correction (proportional to *T*) grows proportionally, remaining a ∼20% addition.
- *Stronger coupling*: If circuit QED parameters evolve toward ultrastrong coupling (g∼1GHz), $\Delta I_{SB}$ could approach one bit, making the strong-coupling correction comparable to the ideal term.

The finite-time correction, due to the long $T_{1}$ of superconducting qubits, remains at approximately 0.55% at 100 ns and 5.5% at 10 ns reset operations. While not necessary for current designs, several strategies could further reduce dissipation:

- *Reversible syndrome extraction*: Measuring ancillas without fully resetting them—using techniques like “warm reset” or coherent feedback—could avoid erasure entirely for some cycles [9]. The thermodynamic cost then shifts from Landauer dissipation to measurement-induced dephasing, which may have different scaling.
- *Reservoir engineering*: Designing the environment to have large thermal conductivity and heat capacity near qubits can mitigate local heating. The phase transition identified in Ref. [12] depends critically on these parameters.
- *Optimal control protocols*: Using geodesic protocols from geometric thermodynamics [13] minimizes the excess dissipation for a given reset time τ, achieving the quantum speed limit bound $Q_{\mathrm{FT}}\approx \hbar /\tau$. This is conceptually aligned with frameworks that classify quantum algorithms by their underlying symmetries [34]. For superconducting qubits, this involves shaping reset pulses to follow thermodynamic optimal paths.
- *Adiabatic reset protocols:* Instead of fast, driven resets, one can use slow, adiabatic passages that return the ancilla to its ground state while keeping the system near equilibrium. Such protocols operate in the quasistatic regime, thereby eliminating finite-time corrections, at the cost of longer reset times. While this approach is not compatible with the fast cycle times required for surface code error correction, it may find application in architectures where speed is less critical or in combination with other mitigation strategies.

Our analysis has focused exclusively on the reset step of the QEC cycle. However, the measurement of ancilla qubits (Step 2) also dissipates energy, often much more than the reset itself. In circuit QED, readout involves sending a microwave pulse through a resonator and amplifying the reflected signal. The energy in the measurement pulse, part of which is dissipated in the attenuators and amplifiers, can be many orders of magnitude larger than $k_{B}T\ln2$. For example, a typical readout pulse contains $10^{4}-10^{5}$ photons at ∼5GHz, corresponding to an energy of $3\times 10^{-20}-3\times 10^{-19}\;J$ per measurement—comparable to or exceeding the total reset heat of $10^{-25}\;J$ per qubit. Moreover, the cryogenic amplifiers used for first-stage amplification dissipate microwatts of power directly at the mixing chamber plate. These contributions, not the fundamental Landauer cost, are the true thermodynamic bottleneck for scalable quantum computing. A complete thermodynamic accounting must include the entire measurement and control chain.

To better allocate thermodynamic costs, we can distinguish three stages in the QEC measurement process [35]:

1. *Reversible premeasurement*: The qubit becomes entangled with a pointer (e.g., a resonator field). This step is unitary and reversible, incurring no heat dissipation. The entropy increase of the qubit is exactly compensated by the pointer’s entropy.
2. *Irreversible record stabilization*: The pointer state is amplified or driven across a threshold to produce a stable classical record. This step dissipates at least $k_{B}T\ln2$ per bit of classical mutual information established between the pointer and the environment.
3. *Memory reset*: The pointer (and sometimes the qubit) is returned to a known initial state. This is the erasure step analyzed in this paper, with cost $Q_{\mathrm{reset}}\geq k_{B}T\ln2+\mathrm{corrections}$.

In typical circuit QED readout, stages 2 and 3 are combined, making it difficult to isolate the dissipation. Recent experiments using nonperturbative cross-Kerr coupling have achieved nearly quantum-limited readout where measurement-induced dissipation is minimized [23]. For such optimized protocols, the dominant cost shifts from stage 2 to stage 3 (reset), which our paper quantifies. Future work should separately characterize each stage to design lower-dissipation QEC cycles.

Our unified framework raises several questions for future investigation:

1. *Interplay of corrections*: Are finite-time and strong-coupling corrections additive, or do they interact nonlinearly? The geometric thermodynamic approach of Ref. [13] naturally incorporates strong coupling, suggesting the two corrections are not independent.
2. *Nonequilibrium environments*: Real cryogenic environments are not perfect thermal baths. How do non-Markovian effects modify the bounds?
3. *Collective effects*: Resetting many ancillas simultaneously might achieve sub-extensive scaling through cooperative phenomena, e.g., by using a common bath or mediated interactions that allow heat to be drained more efficiently than the sum of individual resets [36]. Could such collective reset protocols be implemented in superconducting circuits without introducing additional crosstalk errors?
4. *Experimental verification*: Direct measurement of heat dissipation during rapid ancilla reset in superconducting circuits would test our predictions. Recent advances in nanocalorimetry [37] make such experiments plausible.
5. *Extension to other qubit platforms*: Our analysis focused on superconducting qubits. How do the corrections scale for trapped ions, spin qubits, or photonic systems, where temperatures, coupling strengths, and reset mechanisms differ?
6. *Assessing higher-dimensional correlations:* For systems such as with photonics, hyperentanglement and hybrid entanglement in degrees-of-freedom such as polarization, path, and frequency can play a big role [38,39]. This may enable novel error-correction schemes whose thermodynamic costs remain unexplored.

Resetting ancillas individually yields a total heat load proportional to $N_{\mathrm{phys}}$. However, collective reset protocols—where multiple qubits are coupled to a common dissipative mode—can achieve sub-linear scaling [40,41]. For a register of *N* qubits coupled symmetrically to a single lossy resonator, the time to reset the entire register can scale as $\tau_{\mathrm{reset}}\propto \log N$ under optimal control. It has been conjectured that the total heat dissipation might scale sub-linearly, potentially as $k_{B}T\ln N$ for highly correlated states, but this remains unproven and is not generally valid for the surface code where ancilla states are uncorrelated. This is reminiscent of the sub-extensive work cost for resetting a many-body system with strong correlations. For the surface code, ancilla states are not fully correlated, so the benefit is limited. Nevertheless, for certain QEC codes or for resetting entire logical qubits, collective protocols could reduce the thermodynamic overhead by an order of magnitude or more. Experimental implementation in superconducting circuits remains an open challenge due to crosstalk and fabrication constraints. This paper bridges two previously separate areas of research: the thermodynamic limits of fault tolerance [12] and finite-time quantum thermodynamics beyond weak coupling [13]. By deriving a generalized bound and applying it to concrete architectures, we provide a quantitative framework for assessing the thermodynamic feasibility of large-scale quantum computing. Our conclusion—that thermodynamic costs are manageable—should not be misinterpreted as diminishing the importance of these corrections. Rather, it demonstrates that the fundamental physics allows scalable quantum computing, shifting the focus to engineering challenges of heat removal and temperature stabilization. The dynamical phase transition identified by Bilokur et al. [12] remains relevant: while absolute heat loads are small, the ratio of heating rate to cooling rate determines stability. Our corrections modify the heating rate slightly but do not change the qualitative phase diagram.

Recent advances offer concrete pathways to reduce the dominant heat load from control electronics.

- Adiabatic Quantum Flux Parametron (AQFP) logic achieves power dissipation as low as 81.8 pW per qubit at 4.2 K [42], orders of magnitude lower than conventional cryo-CMOS (often 10–100 mW). Scaling this to $10^{6}$ qubits gives ∼0.08 mW, well within cooling budgets.
- Traditional Josephson traveling-wave parametric amplifiers (JTWPAs) have demonstrated over 20 dB gain [43], while more recent Floquet-mode TWPAs achieve similar gain with near-quantum-limited noise and significantly reduced power dissipation [44]. Although cryogenic HEMT amplifiers dissipate 10–100 μW, Floquet TWPAs are expected to consume much less power, making them promising for large-scale qubit readout.
- Integrated on-chip circulators and isolators using frequency conversion and delay can eliminate bulky ferrite components and reduce heat leakage from control lines [45].

These technologies, combined with the thermodynamic framework developed here, suggest that scalable quantum computing is within reach if engineering efforts focus on low-dissipation control and readout. The analysis of reset thermodynamics for standard QEC cycles could be extended to more complex resource states, such as quantum states that exhibit nested entanglement patterns [46] and may have different entropic costs, or even to encompass aspects of integrated quantum learning paradigms [47].

## 6. Conclusions

We have developed a unified thermodynamic framework for fault-tolerant quantum computing that incorporates finite-time and strong-coupling corrections to the Landauer bound. Our generalized inequality, $Q_{\mathrm{reset}}\geq k_{B}T\ln2+k_{B}T\Delta I_{SB}+O\left(\tau^{-1}\right)$, captures the additional dissipation incurred in realistic QEC implementations. Applying this bound to superconducting qubit architectures, we find that finite-time corrections are negligible for typical qubit parameters $\left(T_{1}∼100\;\mu s\right)$, contributing approximately 0.55% at 100 ns and 5.5% at 10 ns reset operations. Strong-coupling corrections, estimated from residual system-bath mutual information after measurement, add approximately 20% to the ideal cost.

## Data Availability

The data presented in this study are available on request from the corresponding author.
